# Emerging human rights standards on obstetric violence and abuse during childbirth

**DOI:** 10.1002/ijgo.70174

**Published:** 2025-05-01

**Authors:** Camilla Pickles

**Affiliations:** ^1^ Durham Law School Durham University Durham UK; ^2^ Oliver Schreiner School of Law University of the Witwatersrand Johannesburg South Africa

**Keywords:** childbirth, disrespect and abuse, human rights, mistreatment, obstetric violence, safe motherhood, sexual and reproductive health

## Abstract

This article considers emerging human rights standards relevant to obstetric violence and abuse during childbirth in healthcare facilities. It examines the evolution of “safe motherhood” from a focus on physical safety to the recognition of respectful and dignified care as a fundamental right. The analysis traces key developments from WHO, UN Special Rapporteurs, and landmark decisions from human rights bodies like the Committee on the Elimination of Discrimination against Women and the Inter‐American Court of Human Rights. These decisions define obstetric violence as a form of gender‐based discrimination, clarifying state obligations to prevent and address it through legislative reforms, training, effective complaint mechanisms, and remedies for victims. The present article emphasizes free and informed consent, addressing structural inequalities, and promoting respectful maternity care to protect women's reproductive rights.

## 1 INTRODUCTION

Maternity poses significant health burdens for women and girls, encompassing both biological and social dimensions that can lead to serious harms, including death.^1^ However, it is now universally accepted that these outcomes are almost entirely preventable because broader discrimination and political inaction render maternity unsafe for women and girls, especially those from rural, developing, or marginalized communities.^2^ To date, considerable progress has been made in addressing concerns specific to women's reproductive health, with a particular focus on ensuring women's human rights to safe motherhood.^3^

This article considers emerging human rights standards relevant to safe motherhood in the context of facility‐based childbirth, tracing how concerns regarding the right to adequate and quality care have now led to a growing body of human rights jurisprudence to address obstetric violence. It will explore how “safe motherhood” has evolved from a focus on physical safety to encompassing respectful and dignified care that is free from discrimination and violence. The article then examines the international recognition of obstetric violence as a human rights violation, analyzing key developments from WHO, UN Special Rapporteurs, and decisions from human rights bodies like the Committee on the Elimination of Discrimination against Women and the Inter‐American Court of Human Rights (IACtHR). By examining these legal and normative advancements, the article aims to chart emerging state obligations to prevent and address obstetric violence, ultimately contributing to a more comprehensive understanding of women's reproductive rights in the context of childbirth.

Although this article is limited to regional and international human rights developments, it is important to emphasize that states are required to transform emerging human rights standards on obstetric violence into national action capable of protecting and promoting women's right to safe motherhood during childbirth. These efforts are starting to take form, for instance, in South Africa through the inclusion of respectful maternity care principles in the National Integrated Maternal and Perinatal Care Guidelines for South Africa^4^ and evidenced in the growing number of local respectful maternity care training packages for healthcare professionals.^5^

## 2 SAFE MOTHERHOOD: AN EVOLVING HUMAN RIGHTS FRAMEWORK

The notion of “safe motherhood” emerged from the United Nations Safe Motherhood Initiative, which was launched in 1987 by several international human rights agencies to advance the specific goal of addressing the high incidence of preventable maternal mortality and morbidity, with a particular focus on developing countries.^3^, ^6^ The Initiative facilitated the global recognition that preventable maternal death and disability are human rights violations. Subsequently, human rights bodies confirmed the relevance of and clarified the application of several international human rights to the issue of safe motherhood, including the right to life^7^, ^8^ and the right to the highest attainable standard of health.^9^, ^10^, ^11^ Safe motherhood underscores that the right to health includes women's right to maternal and reproductive health, and that states are under positive obligations to actively improve maternal health. They must similarly adopt measures to ensure the provision of accessible, acceptable, and quality pre‐ and postnatal care and emergency obstetric services to prevent foreseeable risk of maternal mortality and morbidity.^10^ Further, failure to provide health care that only women need, such as those required to ensure safe motherhood, is a form of discrimination against women^11^ that governments are obligated to prevent or remedy.^12^, ^13^

In its early formulation within the context of preventable maternal mortality and morbidity, the notion of “safe motherhood” emphasized physically safe progression through pregnancy and childbirth,^14^ in that women were recognized to have an “enforceable right to survive pregnancy and childbirth”.^15^ ^, p. 44^ Safety was deemed achievable through government‐guaranteed access to formal health facilities that are adequately resourced, staffed by skilled medical professionals, and that offer scientifically sound care during pregnancy and childbirth.^15^ However, it is in the wake of the decision of the Committee on the Elimination of Discrimination Against Women in *Alyne da Silva Pimentel v Brazil* ^11^ that a more comprehensive approach to the notion of safe motherhood starts to gain traction.^15^

The case of *Alyne da Silva Pimentel v Brazil* concerned the death of a poor, Brazilian woman of African descent after inadequate treatment for serious obstetric complications at a local health center and the failure of the center to provide timely and effective referral to emergency obstetric care. The Committee established that preventable maternal death constitutes a violation of women's rights to life, health, and non‐discrimination, and it confirmed that states must provide adequate and quality maternity care as part of their non‐discrimination obligations. In finding a violation of Alyne's right to equal access to health care, the Committee applied the WHO guidelines on maternity care, thus endorsing them as the standard by which to determine such violations.^13^ In assessing quality of care, the Committee highlighted technical failures, such as delayed interventions, inadequate medical facilities, and ineffective transfer procedures. Importantly, the Committee also recognized da Silva Pimentel's inhumane treatment as a quality‐of‐care issue, emphasizing how her experiences reflected systemic discrimination against marginalized women based on race, ethnicity, and socioeconomic status.

Significantly, the decision underscores that access to facility‐based maternal care alone is insufficient to achieve safe motherhood. Women continue to face serious harms or avoid accessing facilities due to fear of experiencing disrespect, abuse, or mistreatment.^15^, ^16^ The case emphasizes the need for a comprehensive approach to quality of care that respects women's rights during maternity care provision. As confirmed by the UN Office of the High Commissioner for Human Rights, respectful care for women using health services is a critical dimension of both quality and acceptability elements of the right to health.^17^ ^, p. 3^

## 3 OBSTETRIC VIOLENCE: UNDERMINING SAFE MOTHERHOOD

The emergent international recognition that respectful and dignified maternity care is fundamental to ensuring women's rights to safe motherhood facilitated the international acknowledgment of wide‐spread mistreatment and violence against women during facility‐based childbirth.^16^ Women's experiences included physical and verbal abuse, humiliation, coercive or unconsented medical procedures, lack of confidentiality and violation of privacy, failure to get fully informed consent, refusal to give pain medication, refusal of admission to health facilities, neglect leading to avoidable complications, and detention of women due to an inability to pay hospital bills related to maternity services.^16^ Current research reveals that disrespect and abuse are entrenched features of facility‐based childbirth,^18^, ^19^, ^20^ instigating targeted and high‐level human rights action directed at addressing the issue.

In lieu of a universal charter or instrument to specifically delineate how human rights are implicated in childbirth, WHO issued a groundbreaking statement emphasizing that disrespect and abuse during facility‐based childbirth are violations of women's fundamental rights protected by established international human rights instruments, specifically highlighting women's rights to life, health, integrity, and freedom from discrimination.^16^ It clarified that the right to the highest attainable standard of health includes the right to dignified, respectful health care, and the right to be free from violence and discrimination, with respectful maternal care being categorized as an “essential component of quality of care.”^16^ ^, p. 2^ In a joint statement relevant to women's health rights, international and regional human rights experts recognized that women's right to dignified and respectful care during facility‐based childbirth is undermined by violence against women, harmful gender stereotypes, and multiple and intersecting forms of discrimination.^21^ Consequently, the experts emphasize that if governments are to advance women's rights to safe motherhood during childbirth they must “address acts of obstetric and institutional violence”.^21^

The framing of undignified, disrespectful, and poor quality of maternal care as a particular form of gender‐based violence against women—“obstetric violence”—reflects the already well‐established position in the Americas.^22^, ^23^ Latin American grassroots reproductive rights activists have framed facility‐based abuse as obstetric violence to highlight its connection to the broader pattern of violence against women and systemic inequalities based on gender, race, and socioeconomic status.^15^ This perspective reveals significant overlap between disrespectful and abusive maternity services and wider societal violence against women, specifically emphasizing the underlying intersecting grounds of discrimination towards women in maternal health care that lead to unnecessary suffering and harm. Consequently, women's childbirth experiences were included in broader conversations about violence,^15^ implicating governments' obligations to address obstetric violence under CEDAW^24^ and the Inter‐American Convention on the Prevention, Punishment, and Eradication of Violence against Women (Belém do Pará Convention).^25^, ^26^

Although various human rights conventions aimed at ensuring women's equality do not specifically mention obstetric violence, advocacy efforts to include it within their scope have received resounding support from human rights bodies and experts.^26^

The Special Rapporteur on violence against women, its causes, and consequences recognizes that obstetric violence is a violation of women's rights to health during childbirth and explicitly situates systemic poor quality of care within the remit of the Declaration on the Elimination of Violence against Women and CEDAW.^27^ The report contextualizes obstetric violence within the broader “continuum of violations”^27^ ^, p. 5^ against women rooted in structural inequalities, discrimination, and patriarchal norms. Consequently, state non‐discrimination obligations are applicable to all forms of violence during childbirth and states have an immediate obligation to eliminate discrimination and violence in maternity services through all practical measures, regardless of economic, cultural, or religious constraints. Further, states will remain accountable for private actors who violate women's rights while providing public services, including maternity care.^27^

While the report recommends that states prosecute perpetrators of obstetric violence and provide reparations and compensation to victims, important structural interventions are recommended too. For instance, states are obligated to devote the maximum available resources to ensure that women's health needs are met during childbirth. This requires states to develop national strategies to ensure respectful maternal care services that are aligned with women's international human rights. States must ensure that maternity facilities are adequately resourced, that healthcare professionals are suitably qualified and educated on women's human rights during childbirth, and that accessible and effective complaint mechanisms are available to women. Allegations of obstetric violence and related structural and systemic causes must be investigated with a view to offering adequate redress and to revise laws, policies, and national action plans relevant to women's reproductive health.

Notably, the report identifies the promotion of informed consent and refusal as a key intervention to ensure respectful maternity care and prevent obstetric violence. Informed consent is not merely a procedural requirement concluded with a signature, but a fundamental right to an individualized process of ongoing communication, linked to women's autonomy, dignity, and the right to freedom from coercion. Therefore, states must ensure that health systems function in ways that allow for the provision of clear, comprehensive, and accessible information about proposed care plans, ensure that women understand the information provided, and respect women's decisions without coercion or pressure from hospital staff, relatives, or the broader community. Any law, policy, or practice that requires spousal or third‐party consent discriminates against women and states must adopt laws and policies ensuring effective implementation of patients' free and informed choice of care in maternity services.

The report provides pivotal guidance that was previously missing from the international human rights landscape. Indeed, the report's endorsement by the CEDAW Committee and the IACtHR elevates the significance of its contribution, and the growing body of jurisprudence on obstetric violence confirms and develops the report's recommendations.

### 3.1 State accountability for obstetric violence under CEDAW

The CEDAW Committee's landmark decision in *SFM v Spain* ^28^ addressed obstetric violence as a form of gender‐based discrimination against women during facility‐based childbirth. The Committee recognized that SFM was subjected to medical interventions that were contrary to evidence‐based protocols and performed without her free and informed consent. The Committee noted the Special Rapporteur's framing of obstetric violence as a form of gender‐based violence against women during facility‐based childbirth and recognized that SFM's mistreatment during childbirth was a form of discrimination. It documented that gender stereotypes in health care and medical paternalism frustrated necessary processes that support free and informed decision making, which undermined SFM's autonomy. Further, the Committee made the crucial link between the failure to obtain fully informed consent during childbirth and discrimination against women.

The Committee confirmed that informed consent is required for invasive treatments, and it underscored the importance of providing women with adequate information at every stage of childbirth to enable informed decision making. The Committee also reinforced the principle that consent must be provided freely and before interventions thus ensuring that women's right to participate in decision making is meaningfully supported. Conversely, it ordered that informed consent was not required in emergency situations “where the life of the mother and/or the baby is at risk,”^28^ ^, para 8(i)^ a questionable position that the Committee quickly rectified in its second decision on obstetric violence^29^, ^30^ considered in more detail below. Critical of the Spanish judicial process, the Committee found it perpetuated discriminatory gender stereotypes about women during childbirth, leaving SFM without access to justice. Stereotyping was particularly evident in the judiciary's deference to medical authority, its problematic assumptions about women as passive participants in their childbirth processes, its failure to give equal weight to evidence presented by SFM, and minimizing the harms experienced by SFM during her clinical encounter.

In addition to individual reparation, broader systemic reforms were recommended. The Committee recommended, as part of women's human right to safe motherhood, that the right to free and informed consent be protected and promoted by ensuring the provision of adequate and timely information throughout the childbirth process. This implicates individual healthcare professionals' actions but also emphasizes state responsibility to ensure the possibility for informed consent, provide training for medical professionals on women's reproductive health rights, and conduct research into obstetric violence to inform guidance and public policies to combat it. In addition to recommending that Spain ensures adequate remedies to victim‐survivors, the Committee recommended training judicial and law enforcement personnel on issues relevant to women's reproductive health rights and obstetric violence.

Obstetric violence was brought before the CEDAW Committee again in *NAE v Spain* ^29^ and the Committee's decision marks an evolution in its approach to obstetric violence as a human rights issue. The Committee explicitly recognized that NAE's treatment during childbirth, including non‐consensual medical interventions, denial of critical information, use of her body for student training without consent, and subjection to derogatory attitudes, amounted to obstetric violence. Drawing from sources such as the Special Rapporteur on violence against women's thematic report, the Committee identified specific actions constituting obstetric violence, including early induction of labor without consent, multiple vaginal examinations, denial of food, infantilization, non‐consensual cesarean section performed by residents, separation from the baby, and imposed bottle‐feeding.

Significantly, the Committee clarified its position on free and informed consent, affirming that it is required for all treatments during childbirth, including emergency interventions. This clarification upholds women's decision‐making powers even in high‐risk situations and challenges existing power imbalances between healthcare professionals and birthing women in institutional settings.^30^ The Committee's recommendations, while similar to those in *SFM v Spain*, go further by calling for the establishment, publication, and implementation of a Patients' Bill of Rights, reinforcing the human rights approach to addressing obstetric violence.

Although the cases against Spain are focused on state obligations to exercise due diligence in the administrative and judicial procedures after complaints of obstetric violence, the decisions represent a significant development in the international human rights framework relevant to safe motherhood. The Committee clearly positioned obstetric violence within structural patterns of discrimination against women in healthcare settings, which supports the recognition that obstetric violence is not merely isolated incidents of poor medical practice but a gendered human rights violation that requires international accountability mechanisms. By examining obstetric violence through the lens of CEDAW, the Committee confirms that obstetric violence falls within the remit of the Convention and the obligations that arise from it. This approach strengthens accountability mechanisms by connecting maternity care directly to fundamental human rights principles and ultimately expands the scope of reproductive rights beyond traditional concerns about access to contraception and abortion to include the quality and dignity of care during childbirth. Finally, the cases contribute to developing standards for appropriate maternity care by detailing specific practices that constitute obstetric violence and violations of rights, including treatment outside of the parameters of clinical guidelines, non‐consensual interventions, separation of mother and infant without medical necessity, and interference with breastfeeding. These aspects help establish clearer boundaries for acceptable practice and state obligations in maternity care.

### 3.2 Regional developments

Obstetric violence is yet to be considered in the African human rights system,^31^ but is it increasingly recognized as a form of violence against women and explicitly linked to broader patterns of gender‐based discrimination in other regions. For instance, the Council of Europe Parliamentary Assembly^32^ recognized concerns of obstetric violence in Europe and draws from the Convention on Preventing and Combating Violence against Women and Domestic Violence^33^ to reiterate states' obligations to prevent all forms of violence against women, including obstetric violence. More recently, building from the evolving international recognition of obstetric violence, the European Parliament resolution on Sexual and Reproductive Health and Rights^34^ recognized that obstetric violence is a human rights concern that falls within the European legal framework, and it establishes important connections between women's reproductive rights, bodily autonomy, and protection from violence in health care. Although not binding, the resolution elevates obstetric violence to a human rights issue in the region and provides critical guidance for the development of law and policies relevant to obstetric violence and reproductive rights in the European Union. Despite this significant development, the Inter‐American Human Rights system provides the longest standing and most comprehensive human rights approach to obstetric violence to date.

In 2012, the Committee of Experts of the Follow‐up Mechanism to the Belém do Pará Convention (MESECVI)^35^ affirmed that the Convention encompasses all forms of violence against women, including obstetric violence, recommending its criminalization. Although the obligation to criminalize obstetric violence remains, the IACtHR has expanded states' responsibilities in relation to obstetric violence by integrating broader human rights frameworks.

The case of *Brítez Arce v Argentina* ^36^ concerned a pregnant woman with a history of known risk factors who was admitted to a public hospital after an ultrasound confirmed fetal death. Despite clear indicators of a high‐risk pregnancy, medical staff failed to adequately assess her condition or provide sufficient information regarding the risks and alternatives associated with inducing labor. During labor induction, Brítez Arce was subjected to prolonged neglect, left sitting in a chair for 2 h without appropriate care or support. She experienced severe emotional distress and anxiety from the stillbirth of her son and died soon thereafter. The IACtHR found that the clinical management of her obstetric emergency contributed to her death, citing inadequate information provision, failure to address underlying risk factors, and neglectful treatment during labor. The Court emphasized states' obligation to provide adequate, specialized, and differentiated health services for women during pregnancy, childbirth, and postpartum to prevent maternal mortality and morbidity. It stressed the importance of access to accurate, unbiased information based on scientific evidence as a crucial component of the right to health.

The Court found that Argentina violated Brítez Arce's rights to life, health, and personal integrity under the American Convention on Human Rights. Specifically, the Court determined that Brítez Arce was subjected to obstetric violence, which it defined as a form of gender‐based violence caused by those responsible for women's care at health institutions during pregnancy, childbirth, and postpartum. This violence is expressed through dehumanized, disrespectful, abusive, or negligent treatment, denial of treatment and complete health information, forced medical procedures, and pathologization of natural reproductive processes. This definition is especially significant because it recognizes both acts of commission and omission, acknowledging that violence can occur through direct mistreatment as well as neglect or through withholding information. Thus, the Court's conception of obstetric violence encompasses a spectrum of practices that violate women's dignity and bodily autonomy during reproductive health care. The Court emphasized that states have an obligation to prevent, punish, and abstain from practicing obstetric violence, recognizing women's right to live free from violence.

This groundbreaking decision establishes a clear framework for addressing obstetric violence as a human rights issue, emphasizing state responsibility in ensuring respectful, informed, and quality care for women during pregnancy and childbirth. Indeed, through a progression of cases, the Court has systematically expanded and refined its understanding of obstetric violence, moving from initial recognition to comprehensive application across diverse factual scenarios.

The case of *Maria v Argentina* ^37^ reaffirmed the obstetric violence framework established in *Brítez Arce v Argentina*, applying it to the case of a vulnerable adolescent girl who was denied parental support and access to information during childbirth and was later coerced by hospital staff into relinquishing her son for adoption. The Court, drawing from CEDAW, deemed this treatment “harmful practices” rooted in sex, gender, and age discrimination, adversely impacting the quality of care provided to Maria during childbirth. By recognizing the circumstances as obstetric violence, the Court emphasized the State's duty under the Convention of Belém do Pará to prevent and eradicate gender‐based violence within reproductive health services. *Rodriguez Pacheco v Venezuela* ^38^ clarifies that states have an obligation to establish timely, adequate, and effective reporting mechanisms that recognize obstetric violence as a form of violence against women, and allegations should be investigated with due diligence, perpetrators punished, and victims provided with fair and effective means of compensation.

Most recently, the IACtHR ruled on obstetric violence in the case of *Beatriz v El Salvador*.^39^ Beatriz was diagnosed with health conditions that posed serious health risks during her second pregnancy, and medical examinations confirmed that her fetus had anencephaly. Despite medical recommendations to terminate the pregnancy to protect her health and life, Beatriz was denied a therapeutic abortion due to El Salvador's absolute criminalization of abortion without exceptions, and the Salvadoran courts failed to provide timely or effective guidance, resulting in prolonged delays in the provision of necessary maternal care. After significant deterioration of her health and preventable suffering, Beatriz underwent a cesarean section, and her child survived only briefly after birth. The IACtHR found that denying a woman with a high‐risk pregnancy access to necessary medical care, including abortion, due to criminalization of the procedure, constitutes obstetric violence. The Court found that prolonged delays in care, caused by the criminal law regime, judicial inaction, and lack of protocols for healthcare providers, amounted to dehumanizing treatment. It found that El Salvador violated her right to health and the Court ordered the government to implement directives for the judiciary and medical community on managing high‐risk pregnancies.

An essential contribution from the IACtHR jurisprudence is the Court's authoritative framing of obstetric violence as an intersecting human rights issue in that obstetric violence simultaneously violates multiple human rights. This helps to develop a comprehensive account of state obligations and possibilities for structural reform, in addition to criminalization of obstetric violence. Indeed, given the structural drivers behind obstetric violence and states' obligations to take steps to prevent obstetric violence, it is critical that the framework for accountability extends beyond individual perpetrators to include clear standards for state action and accountability. Finally, given that the Court's human rights analysis was strongly influenced by universal human rights instruments in the establishment of its emerging obstetric violence framework, the Court provides valuable and authoritative insight regarding appropriate human rights responses beyond the Americas.

## 4 CONCLUSION

The evolving human rights framework on obstetric violence marks a significant step towards ensuring women's rights to safe, respectful, and dignified maternity care. From the initial focus on safe motherhood and reducing maternal mortality, the international community has increasingly recognized that quality of care extends beyond physical safety to encompass the right to be free from violence, discrimination, and mistreatment during facility‐based childbirth.

Landmark decisions from the CEDAW Committee and the IACtHR have been instrumental in defining obstetric violence as a form of gender‐based discrimination and a violation of fundamental human rights. These decisions clarify state obligations to prevent and address obstetric violence through various measures, including legislative reforms, training for healthcare professionals and judicial personnel, the establishment of effective complaint mechanisms, and the provision of remedies for victims. The recognition of free and informed consent as a cornerstone of respectful maternity care, along with the emphasis on addressing structural inequalities and discriminatory practices, further strengthens the human rights framework on obstetric violence. As awareness of obstetric violence grows and legal standards continue to develop, it is crucial for states to translate these emerging human rights standards into concrete policies and practices that protect women's reproductive rights and promote respectful maternity care.

## CONFLICT OF INTEREST STATEMENT

The author has no conflicts of interest.

## Data Availability

Data sharing not applicable to this article as no datasets were generated or analysed during the current study.
